# Blockchain as enabling factor for implementing RFID and IoT technologies in VMI: a simulation on the Parmigiano Reggiano supply chain

**DOI:** 10.1007/s12063-022-00324-1

**Published:** 2022-09-23

**Authors:** Antonello Cammarano, Vincenzo Varriale, Francesca Michelino, Mauro Caputo

**Affiliations:** Department of Industrial Engineering, University of Salerno, Fisciano, Italy

**Keywords:** Blockchain, Supply chain, Vendor managed inventory, RFID, Internet of things

## Abstract

Blockchain has recently been associated to Supply Chain Management to solve several problems and change operations management processes. The study proposes to analyse three different scenarios of the Parmigiano Reggiano supply chain considering blockchain technology as an enabler for the use of other technologies such as RFID and the Internet of Things (IoT) and for the exploitation of the Vendor Managed Inventory (VMI) strategy. The study is based on the evaluation of three agent-based simulation scenarios, a traditional "as is" scenario, a second "to be" scenario implementing emerging technologies including blockchain, and a third “to be” scenario that combines the second one with the VMI optimization strategy. The results show how the combined adoption of these technologies improves the procurement process and customer satisfaction. Findings highlight the impacts that the different scenarios have on the supply chain operations in a quantitative way and allows to evaluate the changes in supply chain processes. By employing emerging technologies, order management activities are more automated and time to order and lead time order preparation are reduced. However, to achieve these performances, other data capture tools such as RFID and IoT are needed. Finally, the introduction of the VMI strategy, when enabled by blockchain technology, improves the procurement performances and significantly reduces unfilled orders.

## Introduction

Nowadays, supply chains face several problems in terms of ecosystem complexity (Jaeger et al. 2021; Serdarasan 2013), sustainability (Jaeger and Upadhyay 2020; Sharma et al. 2022; Siddh et al. 2021; Upadhyay et al. 2021a), international logistics (Stojanović and Ivetić 2020), collaboration between the network players (Cammarano et al. 2019; Jraisat et al. 2021; Ralston et al. 2020), demand management (Wang et al. 2015), transparency (Xu et al. 2021), disruptions (Yu et al. 2021) and distortion of information (Xue et al. 2020). These multiple challenges lead companies to constantly innovate and implement systems that use emerging technologies (Agrawal et al. 2022). In particular, one of the purposes of the supply chain is to manage the processes and the transactions between buyers and suppliers, optimizing costs and times but at the same time guaranteeing high quality of the service and the products supplied (Mukhuty et al. 2022). This scenario includes the new Industry 4.0 plans that are based on the connection among new technologies automating different procedures in order to optimize manufacturing and logistics processes of each company in the ecosystem (Li et al. 2021a; Srivastava et al. 2022). Indeed, the effective and efficient supply chain management improves the coordination between supply and demand by reducing costs (Ketokivi and Mahoney 2020). In addition, the challenges of supply chain management have further amplified with the Covid-19 pandemic (Hald and Coslugeanu 2021; Joshi et al. 2022; Sharma et al. 2021). Taking control over data is essential in this scenario as it allows to make decisions that can improve business performance.

Currently, there are several strategies to solve these challenges, such as Collaborative Planning, Forecasting and Replenishment (CPFR) (Singhry and Abd Rahman 2019) or Just in Time (JIT) (Alfayad 2020). There has been an exponential increase in the availability of data and for the implementation of new technological solutions that allow to improve efficient inventory and supply policies (Cammarano et al. 2020). One of the strategies used in this area is VMI which permits upstream actors to manage the goods of downstream players. VMI effectively reduces inventory costs and improves collaboration between buyers and suppliers (Disney and Towill 2003). However, this strategy faces many challenges when collecting data. Some of these challenges include data integrity, accessibility, information delay, data transparency, server centralization, and traceability within the system's stakeholders (Kolb et al. 2018).

To solve these problems, the development and use of emerging technologies is necessary to improve the effectiveness and efficiency of the entire supply chain. For example, data collection can be done by sensors and drones (Sharma et al. 2020). Consequently, by using IoT, this information can be sent to servers for data processing to make more accurate decisions (Lezoche et al. 2020). However, these systems are based on the centralized server-client paradigm which could be easily tampered by hackers (Feng et al. 2020). To solve such huge problems, many scholars are studying the use of distributed ledgers technologies, and specifically blockchain. This technology is based on a distributed and encrypted ledger that allows the transactions to be securely stored and ensures greater transparency between the actors in the chain. Hence, blockchain technology could expand the use cases of VMI (Casino et al. 2019a). There is a high interest of scholars on the combined and connected use of these technologies in the food supply chain (Astill et al. 2019; Lezoche et al. 2020). Several scholars claim that blockchain can enable process management in a more effective and efficient way (Saberi et al. 2019; Upadhyay et al. 2021b; Vu et al. 2021).

However, few works evaluated and compared the efficiency of operations management for the supply chains by integrating or not the blockchain with other technologies. The research focused mainly on improving and measuring the technological performance of blockchain technology—such as latency time, throughput and number of transactions—and less on response times for the supply chain operations (Alonso et al. 2020; Yoon et al. 2020). To be used in supply chain operations, blockchain technology should be driven by external tools and technologies to collect input data. In this way it is possible to carry out concrete, secure, precise and efficient decisions. Current literature little explored the combined effect of blockchain with other emerging technologies to improve the overall supply chain operations. On the one hand, there are no real case studies that combine different technologies on multiple areas of supply chain management; on the other hand, literature focuses on the analysis of single technologies in different areas of the supply chain (Lohmer et al. 2020; Longo et al. 2019; Manupati et al. 2020).

Hence, the purpose of the paper is to evaluate blockchain integrated with other technologies in a food supply chain using a simulation tool. Simulation is a powerful descriptive tool for experimenting, evaluating and comparing different alternatives of new system designs. The key results allow predicting system performance and identifying potential issues. In the absence of a real system model, simulation allows to experiment and compare different alternative models. Moreover, the simulation reproduces new projects avoiding big investments in new systems for which there is a little or no experience, reducing the potential implementation risks. Finally, the simulation models provide numerical and detailed measures of system performance (Carson 2004). Specifically, a comparison is proposed, within the Parmigiano Reggiano cheese supply chain, an excellent Made in Italy product, between a traditional scenario and one with the use of emerging technologies, such as: blockchain, IoT, RFID and smart contracts. Differences in terms of efficiency indicators regarding process times are highlighted. Thereafter, a further scenario is proposed that implements the VMI strategy connected to the use of the blockchain, highlighting further improvements in terms of time performance and customer satisfaction by exploiting the potentials of the shared distributed ledger and smart contracts.

This research addresses the aforementioned gap in both literature and industrial practice of operations management. In order to quantitatively show the advantages that supply chain players can obtain by combining different emerging technologies, the simulation scenarios were developed considering a producer, a delivery company, a wholesaler, three retailers and the customers. The areas and operations considered in the simulation models include all the main organizational, production and delivery processes such as: order management, inventory management and logistics. This article aims to clarify the following research questions:

1. On which supply chain operations can blockchain, IoT, RFID and smart contract derive operational time benefits for organizations?
2. What are the benefits for each participant of supply chain in the various scenarios considered?

The rest of the article is organized as follows: Sect. 2 provides a framework conceptualization of the work. Then, Sect. 3 illustrates a background on blockchain technology and its use in the food supply chain. In addition, the perspective of blockchain technology as an enabler of the VMI strategy is clarified. Section 4 describes the three proposed simulation scenarios, while Sect. 5 shows the output results from the three scenarios. Section 6 discusses the results for the three scenarios and clarifies the practical and managerial implications of the combined use of these technologies. The conclusions will close the work.

## Framework conceptualization

Scientific literature widely highlighted how trust and information sharing are crucial for improving supply chain performances such as flexibility and resilience, especially in an era of globalization (Kasemsap 2017). Trust among players promotes costs reduction and better collaborative relationship among partners (Kim and Chai 2017). In addition, it is important that the actors of supply chains plan production activities and monitor own inventory in a precise, accurate and timely manner. This enables to mitigate the bullwhip effect and reduce issues such as increased inventories, logistics costs and inefficiencies. The phenomenon has significantly reduced with the information and communication technologies (Hofmann 2017; Varriale et al. 2021a). However, when the supply chain expands to other countries, sharing information becomes more complex (Shore 2001). In low-trust scenarios, it is difficult to establish collaborative relationships where data access is essential to efficiently manage supply chain operations (Ebrahim-Khanjari et al. 2012; Michelino et al. 2015). Despite the information technology has reduced information asymmetry, further investments are needed in these fields (Zhong et al. 2016).

Through literature review, conceptual studies, pilot projects and surveys, scientific literature hypothesizes a time reduction for the supply chain operations employing different technologies, included blockchain, without being able to actually verify it since real cases studies are still few. Several authors claim that “stand-alone” blockchain can automate various supply chain operations. For example, Walmart used blockchain to reduce tracking time for its pilot projects (Astill et al. 2019). Casino et al. (2020) analysed a pilot case of a private blockchain that uses smart contact to improve the traceability of dairy products. Other studies focused on improving collaborative and trusting relationships between partners which allows the optimization of information exchange via blockchain (Longo et al. 2019). For example, companies can obtain comprehensive information on the shelf life of food products to manage inventory and plan transportation by reducing waste (Astill et al. 2019; Roeck et al. 2020). Some studies suggest systems for processing complaints to optimize the supply chain resilience among different entities of the network (Kamilaris et al. 2019; Kumar et al. 2020).

**Fig. 1:**
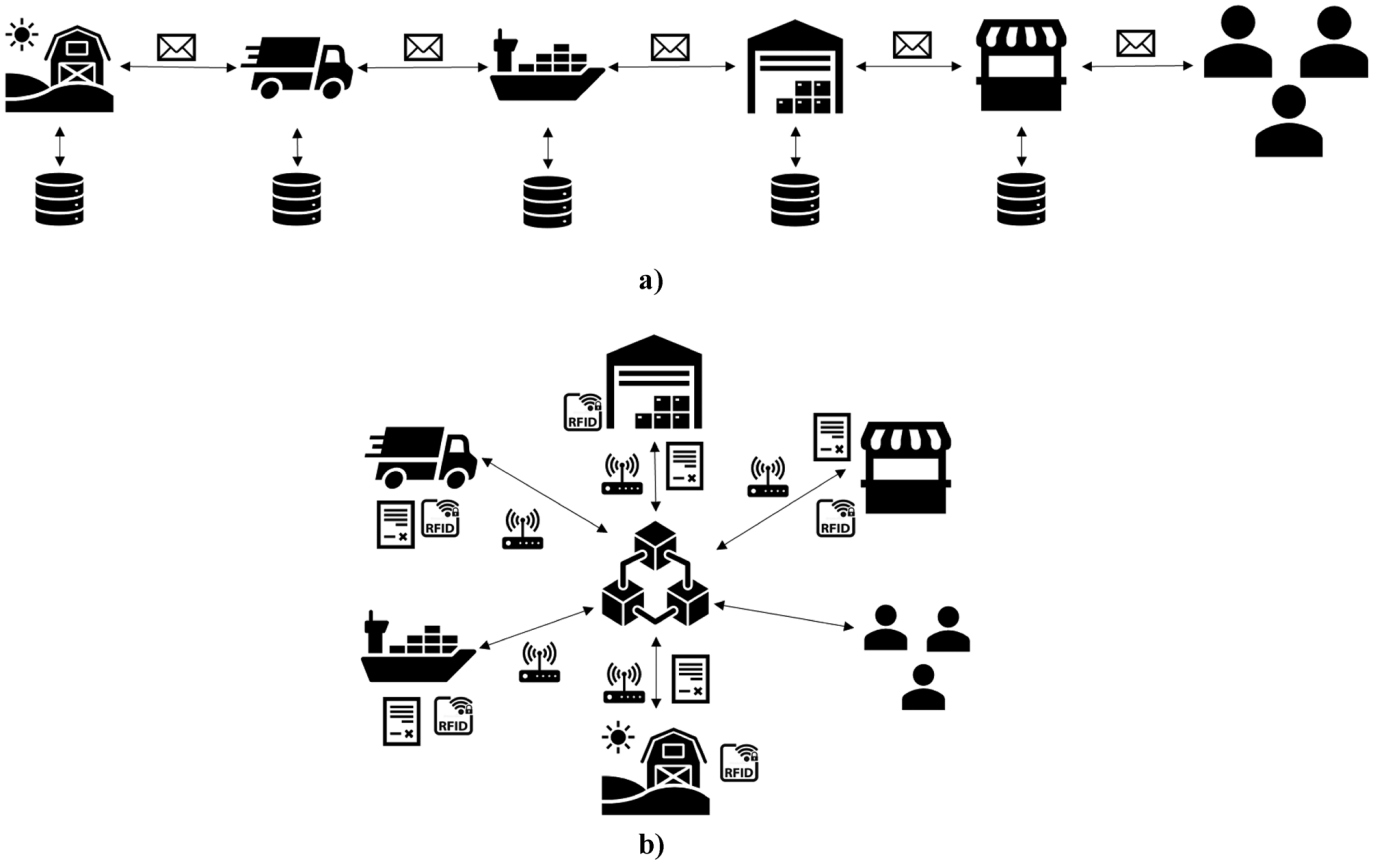
Framework conceptualization of the scenarios considered: **a**) schematization of traditional supply chain; **b**) schematization of emerging technologies integration for the supply chains

Figure 1 shows the framework conceptualization. In scheme a) the partners manage the relationships with the closest actors upstream and downstream. The information is managed internally using the historical data for sales forecasts. Scheme b) provides the integration of different technologies such as RFID, IoT, blockchain and smart contracts for each player. Blockchain becomes an enabler of information sharing by strengthening the resilience of supply chain operations between different actors.

## Literature review

### Blockchain technology

Blockchain technology is a distributed and decentralized ledger that enables data sharing in a transparent and certified way (Choi 2019). The blockchain has had great success in the financial sector with the introduction of the Bitcoin platform (Fosso Wamba et al. 2020). However, its growing focus has also shifted to other areas including the supply chain (Queiroz et al. 2019). Blockchain solutions for supply chains are on the rise as they can improve some of the business and organizational processes (Varriale et al. 2021b). Blockchain technology is presented as an ordered set of blocks containing transactions recorded through consensus mechanisms. Blockchain is tamper-proof: any information recorded in the distributed ledger is unalterable and cannot be changed. The literature divides blockchains into three types: public, private and consortium (Gourisetti et al. 2020). Depending on the information to be shared, the appropriate type is used. In particular, in a public blockchain anyone can connect to the platform and read the stored data (Chang et al. 2020). Instead, in a private blockchain only authorized users can access (Assaqty et al. 2020). Finally, consortium blockchains admit partial authorizations on certain areas for certain users (Qiao et al. 2018). These three types allow different levels of privacy depending on the transactions and information recorded in the distributed ledger. A connected and relevant aspect of the technology is the use of smart contracts, programs that can perform calculations by automating processes and operating as a decentralized machine. Smart contracts are agreements between network participants that can be activated when specific events occur (Christidis and Devetsikiotis 2016). These capabilities are set to improve supply chain management in different steps and processes.

### Blockchain and food supply chain

In recent years, research on the food supply chain has been moving towards innovative technologies and optimization methods (Kamble et al. 2020). The reasons why it is pushing towards innovation in this area are mainly related to risk management (Zhou et al. 2021). The issues affecting the food sector concern: inadequate demand management (Kittipanya-ngam and Tan 2020), incorrect information management (Kouhizadeh et al. 2020), lack of collaboration between the players in the supply chain (Guggenberger et al. 2020) and lack of regulations for controlling food safety and quality (Tse et al. 2018). In addition, more and more consumers demand up-to-date, real-time information on the foods they purchase (Astill et al. 2019). The factors that have greater importance regard the complete transparency and visibility of the supply chain for each participants (Kayikci et al. 2020). Regulatory authorities have imposed standards and certifications on food producers to ensure integrity of food and safety for consumers (Kamble et al. 2020). These certifications improve aspects such as transparency and quality of the food supply chain. However, suppliers, producers, distributors and retailers should exploit alternative approaches, methods and technologies to improve consumer trust in the products purchased (Kittipanya-ngam and Tan 2020). One of the ways to improve these aspects is to move towards a complete digitalization of the entire chain, from the raw materials to the finished product (Bhatti et al. 2021). However, the digitization of a traditional supply chain is a complex and an expensive operation and requires the availability of several resources, both human and digital (Ivanov et al. 2019).

The implementation of IT systems has helped to reduce inventory costs human error and improved the efficiency of communications. Consumer needs regarding the traceability of the entire supply chain are pressing (Hastig and Sodhi 2020). Researchers are investigating tracking and tracing issues, using emerging technologies such as: IoT, GPS, RFID tags and other sensors. These new technologies store information in real time and send it to servers. However, managing data with centralized systems via the IoT has several privacy and security problems (Feng et al. 2020). In this scenario, blockchain technology can be exploited thanks to its peculiarities. The blockchain is the suitable technology to collect data from external devices being a distributed ledger that uses encryption schemes to ensure the security of the system. Since blockchain technology is an immutable ledger, it allows to trace the phases of a product (Pedersen et al. 2019). In addition, another tool that contributes to the overall optimization of the supply chain is the use of smart contracts that can transfer data and information within the blockchain depending on specific conditions. Smart contracts can be implemented to improve order management or to speed up bureaucratic and administrative processes at customs and in international transport (Hasan et al. 2019). Several researchers argue how blockchain can be used in food supply chains to improve process sustainability (Bai and Sarkis 2020; Mukherjee et al. 2021; Upadhyay et al. 2021c; Varriale et al. 2020). Yet, to date main issues of implementing this technology are related to the cost of implementation and the limits of the technological performance (Kouhizadeh et al. 2021).

As shown in Table 1, benefits, challenges and future directions of blockchain have been mainly discussed in the food supply chain sector through systematic literature reviews (SLR). Additionally, scholars have analysed blockchain technology for the food supply chain using manager interviews and case studies. Many articles discuss about the implementation of the technical architecture and how the stakeholders will manage these new digital platforms. However, these changes are still not quantifiable with real data and are difficult to measure for the current state of the art. For example, through a literature review, Vu et al. (2021) show that only 10% of the, sample under analysis concerned documents that quantitatively measured the impacts of blockchain for the food supply chain.

**Table 1:** Studies of blockchain for the food supply chains

#	Authors	Research Method	Sector	Study Nature	Description
1	(Zhao et al. 2019)	SLR	Agri-food	Qualitative and Quantitative	The study analyses 62 academic articles from 2008 to 2018 and identifies four aspects that improve the agri-food supply chain management: traceability, information security, production and sustainable management of water. Additionally, six challenges are identified such as: storage capacity and scalability, loss of privacy, high cost, regulatory issues, speed and latency issues, and lack of skills
2	(Gopi et al. 2019)	SLR	Seafood	Qualitative	The study summarizes the emerging methodologies for determining the provenance and authenticity of seafood. The aim of this review is to provide an overview of the methods that could be used by authorities to enforce regulations and contain risks for the fishing industry to self-regulate and protect from food fraud
3	(George et al. 2019)	Mathematical model	Restaurant	Quantitative	This paper examines the main existing food traceability methods and proposes a restaurant prototype to implement more reliable food traceability using Blockchain and product identifiers. The prototype captures data from various stakeholders along the food supply chain and applies the Food Quality Index algorithm to assess quality performance. The prototype classifies the quality of food for human consumption as well as strengthening the traceability of food
4	(Spadoni et al. 2019)	Case study	Wine	Qualitative	The study is a storytelling of a start-up that adopts blockchain technology to track wine
5	(Kamilaris et al. 2019)	SLR	Agri-food	Qualitative	The article analyses 29 articles and 49 initiatives to outline the state of the art of research and the challenges of adopting the blockchain
6	(Astill et al. 2019)	Conceptual	Food	Qualitative	The article aims to examine technologies for the data management within the food supply chain, such as blockchain and Big Data analysis. In addition, the work considers IoT as a technology that collect data from multiple stages within supply chains and lead to transparent data-driven food production systems
7	(Kamble et al. 2020)	SLR	Agri-food	Qualitative	The article analyses 84 academic articles from 2000 to 2017 proposing an application framework for managers involved in the agri-food supply chain to achieve sustainable performance
8	(Kittipanya-ngam and Tan 2020)	Case study	Food	Qualitative	The article proposes a framework for the digitalization of the food supply chain based on four case studies of Thai companies
9	(Lezoche et al. 2020)	SLR	Agri-food	Qualitative	The article investigates and compares more than one hundred articles on new technologies, including blockchain, to understand the future paths of the agri-food sector
10	(Alonso et al. 2020)	Technical Implementation	Food	Quantitative	The study presents a platform geared for the application of IoT, Edge Computing, Artificial Intelligence and Blockchain in Smart Farming environments, to monitor the status of dairy cattle and forage cereals in real time, as well as ensuring the traceability and sustainability of the several processes involved in production
11	(Klerkx and Rose 2020)	Conceptual	Agri-food	Qualitative	The study presents a theoretical framework on how enabling technologies of agri-food 4.0 can have potential impacts on the agri-food supply chain management
12	(Ciruela-Lorenzo et al. 2020)	Case study	Agri-food	Qualitative	The article provides a review of main digital technologies, such as the Internet of Things, robots, Artificial Intelligence, Big Data and Blockchain, and how these technologies could help decision-making actors. These theories are described through two case studies of agricultural cooperatives in Spain
13	(Feng et al. 2020)	Technical Implementation	Agri-food	Quantitative	The article proposes an architecture design framework and flowchart for blockchain-based food traceability to highlight the benefits and challenges of implementing blockchain
14	(Köhler and Pizzol 2020)	Case study	Food	Qualitative	The study analyses six food supply chain case studies by evaluating four different components of technology: technique, knowledge, organization and product. The study provides new insights into how blockchain can be implemented in food supply chains
15	(Osmanoglu et al. 2020)	Mathematical Model	Agri-food	Qualitative	The study proposes a blockchain-based solution that estimates the yield of agricultural products
16	(Shahid et al. 2020)	Technical implementation	Agri-food	Quantitative	The study proposes technology efficiency solutions to improve information recording performance by exploiting optimization algorithms. In this work, simulations and evaluations of smart contracts are presented along with security and vulnerability analysis
17	(Kayikci et al. 2020)	SLR	Food	Qualitative	The study analyses 125 articles from 2008 to 2020 and investigates the suitability of blockchain technology in solving the main challenges, such as traceability, trust and accountability in the food industry
18	(Della Valle and Oliver 2020)	Interviews	Food	Qualitative	The study features 18 interviews with experienced blockchain managers for supply chains. Analysis shows that blockchain does not appear to be a disruptive technology. Five enablers are presented that can foster rapid blockchain adoption in the industry
19	(Stranieri et al. 2021)	Case study and interviews	Agri-food	Qualitative	The study proposes a conceptual framework that includes performances discussed in literature: efficiency, flexibility, responsiveness, food quality and transparency of supply chains. These dimensions are assessed using a case study. The data were collected through semi-structured interviews with key managers in the different phases of the three supply chains and were systematically analysed through a thematic analysis
20	(Rainero and Modarelli 2021)	Interviews	Food	Quantitative	The article is an exploratory analysis of customer perceptions and real knowledge of the blockchain in the food and beverage sector. The study is based on 80 surveys and interviews
21	(Mishra and Maheshwari 2021)	Conceptual	Food	Qualitative	The study proposes a conceptual framework for the application of blockchain in the Public Distribution System in India to manage the grains supply chain
22	(Vu et al. 2021)	SLR	Food	Qualitative	The study analysis 69 articles to assess the barriers, applications and implementation stages of Blockchain within food supply chains
23	(Sharma et al. 2021)	Interviews	Food	Quantitative	This study provides insights to decision makers, managers to make meaningful decisions during an emergency using blockchain technology via multi-criteria approaches
24	(Bechtsis et al. 2021)	SLR and case study	Food	Qualitative	This article highlights through a literature review and the evaluation of a case study how blockchain technology can improve the security and resilience of the supply chain
25	(Yi et al. 2021)	Interviews	Food	Qualitative	The enabling factors of blockchain are highlighted and understood through the interview of 21 members of the food supply chain in China
26	(Li et al. 2021b)	SLR	Food	Qualitative	The paper analysis the main blockchain platforms used in food supply chains and conducts an analysis to explore the benefits and challenges of the technology
27	(Kramer et al. 2021)	SLR	Food	Qualitative	The research is based on a broad overview of the literature review and exploratory use cases of blockchain implementations in the agri-food industry
28	(Saurabh and Dey 2021)	Conceptual and Technical implementation	Wine	Quantitative	The study proposes what are the factors driving the adoption of blockchain and shows qualitative implementation scenarios for the wine supply chain
29	(Galanakis et al. 2021)	Conceptual	Food	Qualitative	The article theoretically investigates how technologies, including blockchain, can mitigate the effects of the post lockdown of COVID-19
30	(Nurgazina et al. 2021)	SLR	Food	Qualitative	The study analyses 69 articles to understand the effect that blockchain and IoT can have on the sustainability of food supply chains
31	(Tsolakis et al. 2021)	Case study and interviews	Fish	Qualitative and Quantitative	The study proposes the design of blockchain on food supply chains that promote sustainable development goals, in the context of the Thai seafood industry. A possible implementation of blockchain is shown through the analysis of fish case studies
32	(Ali et al. 2021)	Case study	Food	Qualitative and Quantitative	The study proposes the analysis of five case studies to show how certain challenges for the halal food supply chain have been overcome
33	(Yang et al. 2021)	Mathematical model	Food	Quantitative	The study analyses, through game theory, operational decisions and blockchain adoption strategies for a food supply chain consisting of a platform and a supplier
34	(Joo and Han 2021)	Mathematical model	Food	Quantitative	The article examines the features of distributed trust in the blockchain-based food supply chain and tests seven hypotheses using a structural equation model that integrates distributed trust (i.e. transparency, traceability and security) and user satisfaction
35	(Rana et al. 2021)	SLR	Agri-food	Qualitative	The study analyses academic journals from 2010 to 2020 that discuss blockchain applications in the food supply chain to determine future directions
36	(Benyam et al. 2021)	SLR	Food	Qualitative	The study analyses 24 articles to investigate the role of digital agricultural technologies in enabling the prevention / reduction of food and waste loss from a global perspective

Hence, the study aims to measure the impacts that blockchain connected to other technologies has on supply chain operations. Starting from previous studies, that have conceptually designed the different food supply chain configurations through the adoption of blockchain and other technologies without demonstrating their possible effects on supply chain operations, the study aims to measure what are the long-term impacts on time performances and relative customer satisfaction that these technologies carry out for a food supply chain.

### The combination of VMI and blockchain

VMI strategy has been extensively studied in literature (Salem and Elomri 2017). Several authors focused on the success factors for its effective implementation, such as the exchange of information and collaboration between the actors in the chain (Ryu 2016). VMI is used to reduce the bullwhip effect (Disney and Towill 2003), manage orders (Yao et al. 2010), improve the service level (Shi and Xiao 2015), reduce costs (Zhang et al. 2007) and reduce the inventory (Lee et al. 2016). Obtaining precise information on the demand in advance would allow the seller to respond quickly to unexpected orders and improve supply planning (Dong et al. 2014). Furthermore, VMI allows to stabilize the frequency of purchase orders as the seller has a complete knowledge of the downstream demand (Taleizadeh et al. 2015). On the one hand, VMI encourages collaboration with other players in the supply chain, on the other hand, shared knowledge could harm the other players in the chain (Disney and Towill 2003). Currently, the information exchange with traditional technologies could be tampered with, corrupted and not certified. The actors in the supply chain could have opportunistic behaviours or they can make mistakes such as misalignments of real inventory and IT inventory (Kamilaris et al. 2019). For this reasons, blockchain technology would more easily enable the VMI strategy. Indeed, the use of blockchain technology and smart contracts for VMI operations would reduce the need for human intervention (Omar et al. 2020). Features such as data integrity, security and immutability help to trust the mechanism in which the technology works rather than the relationships between network partners (Pedersen et al. 2019). In this way, actors operating with blockchains can trust the information stored in the distributed ledger and they can plan order management (Casino et al. 2019b). Thus, the blockchain enhances the principles of VMI by allowing complete data sharing, traceability and transparency (Guggenberger et al. 2020). Making this information available to those who manage the inventory upstream will enable them to accurately manage the demand by frequently monitoring the stock level. In this way, buyers will place more frequent and smaller orders and consequently have cost savings. At the same time, blockchain will ensure better collaboration between stakeholders as the secure bidirectional exchange of data will increase trust between parties (Chang et al. 2020). Using blockchain, the producer can access to the data shared by wholesalers and retailers. It allows to identify exactly when their downstream customers have reached the reorder level. The combination of the VMI strategy with blockchain technology creates an agile and responsive supply chain. Some researchers are investigating the binomial blockchain and VMI (Kolb et al. 2018). Some propose solutions from a conceptual and theoretical point of view (Casino et al. 2019b). Others suggest solutions in which they show the mechanisms of smart contracts and the security performances of the system (Omar et al. 2020). This article differs from the others because the aim of the research is to evaluate *ex-ante* the possible impact that combination of emerging technologies could obtain on the traditional business processes operations of a food supply chain. The purpose of the article is to evaluate how the visibility of the chain by the actors, the information sharing and the management of integrated emerging technologies can have an impact on the operational supply chain performance.

## Research methodology

In the last five years, research on blockchain for supply chain management has increased exponentially, however few papers have evaluated the performance of the technology in managing supply chains operations. Exploratory research based on case studies allows a better understanding of the phenomenon that can lead to the development of new theories through processing (Gehman et al. 2018). The study of Parmigiano Reggiano cheese production and distribution case is fundamental for easy data access and the close proximity of the phenomenon (Eisenhardt 1989). Simulation provides an experimentation platform that emulate real conditions through a dynamic set of objects and variables (Weick 1989). Table 2 reports a list of simulation works considering the blockchain adoption for supply chains. From this table, the articles that highlight the potential of blockchain within supply chain processes measuring its benefits and disadvantages are relatively few, despite how much this topic has been discussed in the last period. Few works have assessed the impacts that blockchain, alone or integrated with other technologies, could carry out in terms of improving processes within supply chain operations. Previous studies evaluated technology challenges such as scalability, privacy, and security issues (Alonso et al. 2020; Omar et al. 2020; Shahid et al. 2020), others on costs issues (Longo et al. 2019; Manupati et al. 2020; Tozanlı et al. 2020a). Some authors analysed time performances, but only on single case studies and in specific areas of order management (Martinez et al. 2019). Others have used the times to evaluate how the volatility of demand fluctuates and how much stock is needed for the actors in the network (Lohmer et al. 2020). Our work differs from the others because it evaluates the times on all the operations processes of the Parmigiano Reggiano supply chain for all the actors involved. Furthermore, our work evaluates ex-ante the promising impacts that blockchain, IoT, RFID and VMI could have on the Parmigiano Reggiano supply chain. This work aims to explore, quantify and test what the literature has promoted in this research area in recent years. The ability to simulate and perform a cost–benefit analysis is essential to gain adoption by more participants and not just by multinationals where profits are greater.

**Table 2:** Simulation studies of blockchain for the supply chains

Reference	Other technologies/strategies	Area	Sector	Topic	Software	Technological Performance of Blockchain						Operations Performance of Blockchain for the supply chains				
						Smart contract	N. of transactions	Architecture cost	Speed of transactions	Time consuming	Transaction costs	Time performance	Costs	Stock performance	Demand volatility	Carbon tax
(Dasaklis and Casino 2019)	RFID, IoT, VMI	Inventory management	Manufacturing	Inventory strategy	Ethereum	x										
(Omar et al. 2020)	VMI	Inventory management	No declared	Inventory strategy	Ethereum	x			x		x					
(Helo and Shamsuzzoha 2020)	RFID, IoT	Logistics	Manufacturing	Shipping, tracking & tracing	Ethereum	x										
(Yoon et al. 2020)	No other technologies	Logistics	Manufacturing	International trades	No declared (Mathematical models)		x								x	
(Longo et al. 2019)	No other technologies	Inventory management	No declared	Information sharing	Unicalcoin and Java			x	x		x		x			
(Hasan et al. 2019)	IoT, RFID	Logistics	No declared	Shipping	Ethereum	x										
(Casino et al. 2019a)	IoT, RFID	No specific area	Food	Tracking & Tracing	Ethereum	x										
(Martinez et al. 2019)	No other technologies	Order management	Manufacturing	System architecture & performance	Simul8							x				
(Manupati et al. 2020)	No other technologies	Inventory management	Manufacturing	Shipping and market demand	Matlab					x	x		x		x	x
(Lohmer et al. 2020)	No other technologies	No specific area	No declared	Risk management	AnyLogic 8.5.0							x		x	x	
(Dolgui et al. 2020)	No other technologies	Logistics	Manufacturing	System architecture & performance	Hyperledger Fabric	x										
(Tozanlı et al. 2020b)	No other technologies	Production	High-tech	System architecture & performance	No declared (Discret-Event Simulation)								x			
(Alonso et al. 2020)	Edge Computing, RFID, IoT, QR Code	Production	Dairy farming	System architecture & performance	No declared				x	x						
(Sund et al. 2020)	No other technologies	Logistics	Furniture retailer	Shipping	Quorum	x	x		x	x						
(Shahid et al. 2020)	No other technologies	No specific area	Agri-food	Tracking & Tracing	Remix Integrated Development Environment (IDE) Ganache and Metamask	x	x				x					
(Bai et al. 2021)	No other technologies	Production	Agriculture	Tracking	Phyton 3.5		x									
(Tozanli et al. 2020a)	RFID, IoT	Waste management	Electronic	Disassembly-to-order	Arena v15.1								x			
**This article**	**RFID, IoT, VMI**	**Order management, logistics and inventory management**	**Food**	**Inventory strategy and market demand**	**Anylogic 7.0.2**							**x**		**x**		

**Fig. 2:**
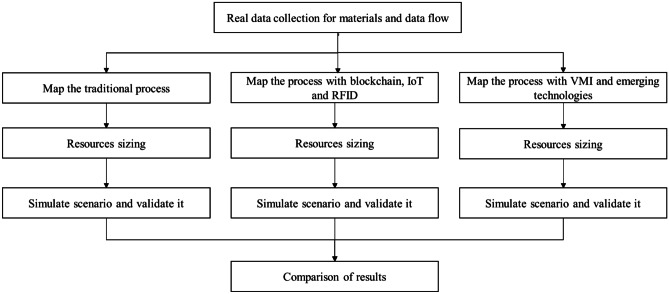
Steps and techniques for the construction of the three scenarios

The simulation approach used is discrete events because it provides a detailed analysis of all phases and is precise, reliable and easy to program. In particular, starting from the most accredited literature on the use of simulation, three scenarios of the cheese supply chain have been developed: a first traditional scenario "as is", without the use of blockchain and other technologies (Bottani and Montanari 2010; Muravev et al. 2019); a second scenario "to be" with the combined use of blockchain technology, IoT and RFID without VMI strategy (Lohmer et al. 2020; Longo et al. 2019; Martinez et al. 2019); and, finally, a third scenario "to be" with the addition of the VMI optimization strategy (Casino et al. 2019b; Omar et al. 2020). Anylogic 7.0.2 Professional was used for simulations2. Figure 2 shows the methods and steps that allowed the construction of the entire architecture.

### Simulation study and data collection

In order to improve the state of the art of the existing literature and validate the work, a simulation study of an Italian factory of Parmigiano Reggiano that exports cheese abroad was considered. In particular, the supply chain was simulated starting from the producer to the final retailer. The phases of warehousing, logistics and order management were considered. In this way it was possible to compare the results and provide a good representation of what blockchain can change. Secondary sources were used for data collection: statistical reports (ISTAT 2022), consortium reports (Parmigiano 2022), scientific articles presented in Table 2 and online reports.

### Design of the simulation study

The following aspects should be specified in a simulation study: input parameters to vary, output parameters, duration of the warm-up phase and execution time of the model and number of replication (Carson 2004). The detailed parameters of our simulation study are shown in Table 3.

**Table 3:** Parameter of simulation experiments

*Model runtime*	0–18 months. The simulation model starts without a warm-up phase as it has been preloaded
*Varying input parameters*	Phases of order management for each actor in the supply chain and phase of loading / unloading of the goods managed by the delivery company
*Output parameters*	There are 12 output parameters divided between the different actors •lead time order preparation for the producer; •shipping time for the delivery company; •time to order for the wholesaler; •Unfilled orders, service level and lead time for each retailer
*Number of runs*	10 replications for each model with relative precision 0.01

### Traditional food supply chain model

**Fig. 3:**
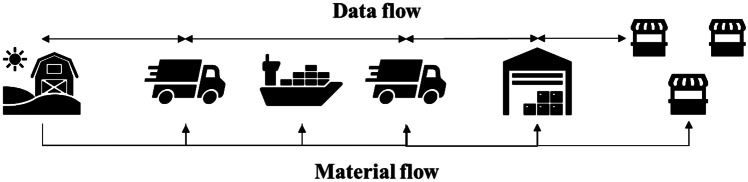
Traditional scenario scheme. The material flow goes from upstream to downstream while the data flow is exchanged by each actor in the supply chain

The network is composed by a producer (P), a delivery company (DC), a wholesaler (W), and three retailers (RA, RB and RC). The network structure and materials flow are presented in Fig. 3. The producer supplies the wholesaler, which, in turn, supplies the three retailers. The network is based on the export of 12 months aged Parmigiano Reggiano from Italy to Spain. The producer is located in Reggio Emilia (Italy), the wholesaler is located in the industrial area of Barcelona and the three retailers at different distances in the metropolitan city of Barcelona.

The daily number of potential **c**ustomers of each retailer changes based on the inter-arrival times modelled with an exponential distribution according to the opening hours of the retailers (see Appendix A). In addition, each retailer divides the cheese wheel into 200 pieces of 200 g and the purchase percentages have been modelled with a triangular distribution. The system was preloaded to eliminate the transitory: in this way the products are already available in stock. In addition to the aging time, the storing, checking, packing and picking times of the producer and wholesaler were also estimated. The wholesaler and the retailers order the products when they reach a limit value, in this way a reorder time is defined. Specifically, RA reorders four cheese wheels if it has a stock level below two cheese wheels after a five-day manual check, while RB and RC reorder three cheese wheels if they have a stock level below two cheese wheels every five days. Finally, the wholesaler periodically replenishes 275 cheese wheels every eight days, as it supplies other retailers that have not been modelled. The shipment from the producer to the wholesaler is based on an intermodal transport (truck-ship-truck) performed by a delivery company. Once the shipment has been designed according to the agreements made and what has been defined, the carrier will go to the producer’s warehouse to load the products. Before proceeding with loading, the carrier checks whether the goods placed in the warehouse shipping zone reflect what is defined in the order. After this operation the goods are loaded, and the seal is affixed. Each seal has a unique identification code which is also reported on the documentation. After applying the seal, the documents that will accompany the goods throughout the shipment are signed. The loading takes place in Full Truck Load mode to minimize shipping costs, therefore the truck load is 275 units. Upon arrival of the goods at the wholesaler, an operator verifies the integrity of the seal, the documentation and the condition of the goods. Following these operations there is the signature of the documents certifying the successful delivery. Any reservations will also be placed on these documents in the event of goods damage. To close the order the documentation must be delivered to the administrative office for the billing. In a traditional process it is necessary to wait that the carrier returns to the delivery company headquarters and delivers the documents. Finally, depending on the orders received by the wholesaler, the goods are shipped to the retailers. From Monday to Friday, a unit of each partner keeps track of data such as stock levels, the quantity of goods sold and the quantity of unfilled orders. Since the orders have been shipped, the stock level must be updated. This process is carried out by an operator periodically at regular intervals of five days. This modelling has been implemented for each player in the supply chain.

Table 4 illustrates the areas, resources and equipment considered in this scenario for each actor with the relative description.

**Table 4:** Area, resources, equipment and activities involved in the traditional supply chain scenario

**Area**	**Activities**	**Resources**	**Equipment**	**Description**	**P**	**DC**	**W**	**RA**	**RB**	**RC**
**Order management**	Order receipt	Order clerk	Excel; e-mail; phone	The order clerk supervises the order management steps. He checks the real availability of the cheese wheels in the warehouse, organizes all the phases and sends the information to the employees who should handle of carrying out the physical operations for the shipping	✓	✓	✓			
	Order processing				✓	✓	✓	✓	✓	✓
	Order generation					✓	✓	✓	✓	✓
**Inventory management**	Storing	Warehouse worker	Stacker crane	Worker that deals with the cheese wheels transportation from the reception area to the stacker crane for loading into the shelving	✓		✓			
	Picking		Forklift	Worker that deals with the cheese wheels transportation for quality control	✓		✓			
	Checking			Worker selected for the quality control of cheese wheels	✓					
	Packing			Worker that deals with both the cheese wheels packaging and the creation of the shipment batch. In addition, he provides the cheese wheels transportation for loading on the truck to be used for the transportation step	✓		✓			
**Logistics**	Checking pallet	Truck Driver	Truck	Driver in charge of checking the quality of cheese wheel packages		✓	✓			
	Loading pallet			Driver in charge of loading goods		✓	✓			
	Signing pallet			Driver in charge of verifying the documentation		✓	✓			
	Shipping			Driver in charge of shipping goods		✓	✓			
	Unloading pallet			Driver in charge of unloading goods		✓	✓			

### Simulation model with blockchain, IoT and RFID within the food supply chain

**Fig. 4:**
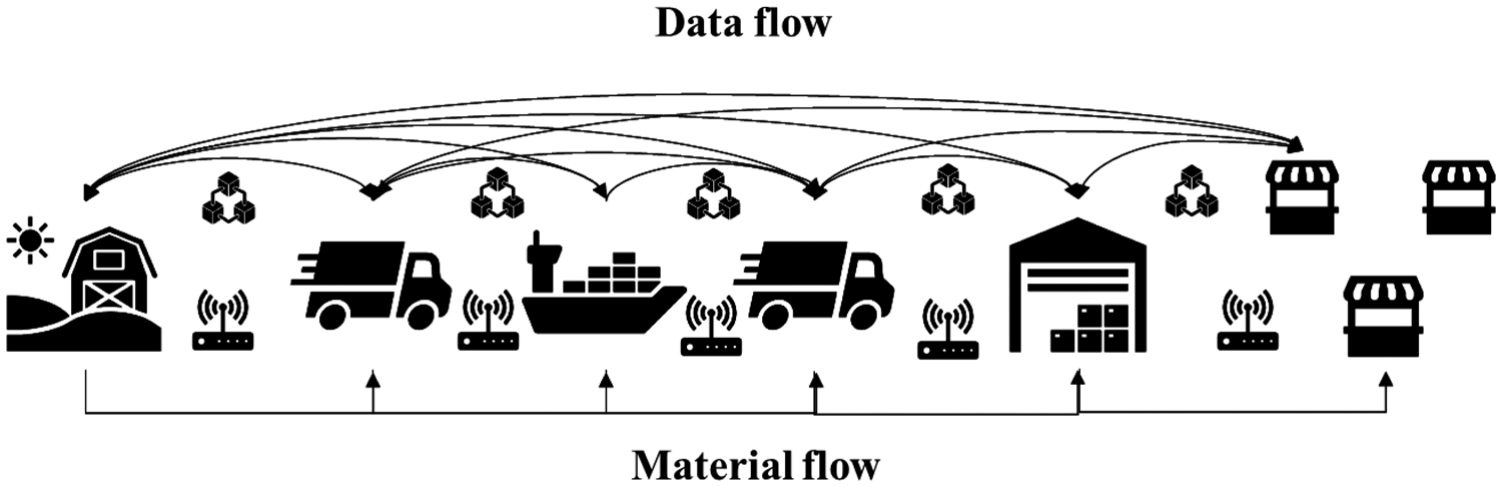
Scenario with the use of technologies such as IoT, RFID and blockchain. The data flow is shared between the partners of the chain using blockchain

The second simulation scenario regards the combined use of emerging blockchain, IoT and RFID technologies. These technologies will allow the real-time control of the storage of products and the transactions that have taken place between the network players. The events are collected by the sensors and are consequently stored within the blockchain. The technologies are installed in the warehouses of each actor and in the truck of the DC in order to constantly monitor every single phase (Fig. 4). The orders management between the actors is automated using smart contracts. The simulation scenario is based on a private blockchain using Hyperledger Fabric (Hyperledger 2022). Private blockchains can be used for the respective relationships between entities. The only advantage of blockchain over a conventional solution that uses other IT methods in this scenario is its immutability.

In this scenario each actor has its key pair for transaction access, depending on its role. The use of these new technologies leads to a constant track of stock levels and the quantities of the goods sold. The stock level is updated in real time. In this case, the alignment and coordination phase of IT and real warehouses is reduced. In particular, the reordering process is not based on a periodic manual check on the quantity available in the warehouse for both retailers and wholesalers. The data monitoring activities are carried out by the RFID sensors and the IoT infrastructure that capture the data constantly. The order management unit will have an alert signal whenever the quantities in the warehouse reach the limit below the pre-established stock quantity, which is the same as in the traditional scenario. Other players, such as the DC, can participate in the smart contract and have permissions to update and change product status. When goods arrive at destination, the receiver checks the smart contract data and accepts them, confirming the transaction. Finally, the product is delivered to the final actor. The status of the goods including location, transport conditions, delivery times and temperature will be updated within the distributed register to keep track of events in real time.

Table 5 illustrates the areas, resources and equipment considered in this scenario for each actor with the relative description.

**Table 5:** Area, resources, equipment and activities involved in the supply chain scenario with IoT, RFID, blockchain and smart contract

**Area**	**Activities**	**Resources**	**Equipment**	**Description**	**P**	**DC**	**W**	**RA**	**RB**	**RC**
**Order management**	Order receipt	Order clerk	Blockchain; Smart Contract	The order clerk supervises the order management steps. He checks the real availability of the cheese wheels in the warehouse using blockchain, organizes all the steps and sends the information to the worker who should handle of carrying out the physical operations for the shipping	✓	✓	✓			
	Order processing				✓	✓	✓	✓	✓	✓
	Order generation					✓	✓	✓	✓	✓
**Inventory management**	Storing	Warehouse worker	Stacker crane; RFID; IoT; Blockchain	Worker that deals with the cheese wheels transportation from the reception area to the stacker crane for loading into the shelving. Inside the shelves there are RFID and IoT infrastructure to monitor the goods in the warehouse	✓		✓			
	Picking		Forklift	Worker that deals with the cheese wheels transportation for quality control	✓		✓			
	Checking			Worker selected for the quality control of cheese wheels	✓					
	Packing		RFID	Worker that deals with both the cheese wheels packaging and the creation of the shipment batch. An RFID sensor is applied to each package. The quality of the batch is tracked via a blockchain transaction. In addition, he provides the cheese wheels transportation for loading on the truck to be used for the transportation step	✓		✓			
**Logistics**	Checking pallet	Truck Driver	Truck; Blockchain	Driver in charge of checking the quality of cheese wheel packages. The driver checks that the packages are guaranteed by viewing the transaction in blockchain		✓	✓			
	Loading pallet			Driver in charge of loading goods		✓	✓			
	Signing pallet		Blockchain	Driver in charge of verifying the documentation through the pair keys		✓	✓			
	Shipping		RFID; IoT	Driver in charge of the shipment of the goods constantly monitored along the route with RFID and IoT infrastructure		✓	✓			
	Unloading pallet			Driver in charge of unloading goods		✓	✓			

### VMI and emerging technologies model within the food supply chain

**Fig. 5:**
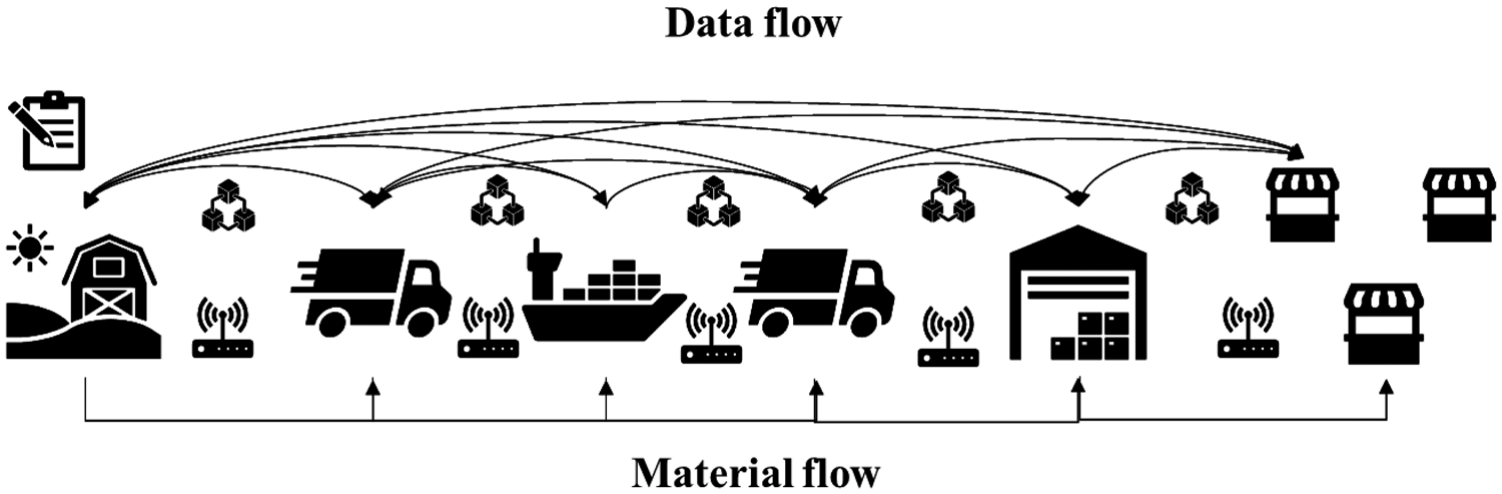
The third scenario concern the VMI strategy combined with emerging technologies. The producer manages the orders downstream thanks to the complete visibility of the blockchain

The third scenario regards the combined use of VMI strategies and digital technologies to obtain a further optimization of the chain. In this case, the wholesaler’s and retailers’ warehouses are managed by the producer. Indeed, using the blockchain technology, the producer could know the storage units of each downstream actor. The retailers and the wholesaler keep an up-to-date track of their inventory through sensors and IoT infrastructure and record these data to the distributed ledger. The producer controls the inventory of the actors via blockchain and, when needed, activates a smart contract with a new order to reload the downstream actors based on previously identified conditions and information (Fig. 5). In addition, the orders management and the activities to be carried out between the players are automated through the use of smart contracts. Also, in this case the reordering process of the cheese wheels for the downstream actors is based on the same previous input data. The actors send their inventory status to the blockchain daily using off-chain storage such as the InterPlanetary File System (IPFS) (Baumgart and Mies 2007; Casino et al. 2019b). Table 6 illustrates the areas, resources and equipment considered in this scenario for each actor with the relative activities description.

**Table 6:** Area, resources, equipment and activities involved in the supply chain scenario with VMI

**Area**	**Activities**	**Resources**	**Equipment**	**Description**	**P**	**DC**	**W**	**RA**	**RB**	**RC**
**Order management**	Order receipt	Order clerk	Blockchain; Smart Contract	The order clerk supervises the order management steps. He checks the real availability of the cheese wheels in the warehouse using blockchain, organizes all the steps and sends the information to the worker who should handle of carrying out the physical operations for the shipping. He oversees the relations with the downstream players in the supply chain network employing a smart contract	✓	✓				
	Order processing				✓	✓				
	Order generation				✓	✓				
**Inventory management**	Storing	Warehouse worker	Stacker crane; RFID; IoT; Blockchain	Worker that deals with the cheese wheels transportation from the reception area to the stacker crane for loading into the shelving. Inside the shelves there are RFID and IoT infrastructure to monitor the goods in the warehouse	✓		✓			
	Picking	Warehouse worker	Forklift	Worker that deals with the cheese wheels transportation for quality control	✓		✓			
	Checking	Warehouse worker		Worker selected for the quality control of cheese wheels	✓					
	Packing	Warehouse worker	RFID	Worker that deals with both the cheese wheels packaging and the creation of the shipment batch. An RFID sensor is applied to each package. The quality of the batch is tracked via a blockchain transaction. In addition, he provides the cheese wheels transportation for loading on the truck to be used for the transportation step	✓		✓			
**Logistics**	Checking pallet	Truck Driver	Truck; Blockchain	Driver in charge of checking the quality of cheese wheel packages. The driver checks that the packages are guaranteed by viewing the transaction in blockchain		✓	✓			
	Loading pallet			Driver in charge of loading goods		✓	✓			
	Signing pallet		Blockchain	Driver in charge of verifying the documentation through the pair keys		✓	✓			
	Shipping		RFID; IoT	Driver in charge of the shipment of the goods constantly monitored along the route with RFID and IoT infrastructure		✓	✓			
	Unloading pallet			Driver in charge of unloading goods		✓	✓			

### Comparison between the three scenarios

In the traditional scenario the order management is based on exchanges of emails, phone calls, the use of Excel and different IT systems. The lack of standardization of the activities requires more time for the practices. The orders between several players are highly manual and consequently there is a cost associated with human resources. Also, due to the laborious manual tasks required for each order, processing and response times are long. In addition, each customer (wholesaler and retailers) sends an order request to the order management unit, then this unit checks the stock in the warehouse, solves any problem and finally approves the order request. Consequently, the order management unit transmits the order specifications to the operators to carry out the delivery. The order management of the scenario with blockchain, RFID and IoT is automated through a smart contract that will allow to carry out the order upon the occurrence of specific conditions depending on the presence of the goods in the warehouse provided by the real-time control via RFID sensors of the inventory. In this case, the use of human resources and process time are reduced as the smart contract was previously implemented in accordance with contractual agreements. However, in the third scenario the order management is further optimized with the VMI strategy. In fact, thanks to the safe and certified visibility of the goods in the inventories of the different players, the producer can activate the smart contract when necessary since he can view all the transactions and know the level of stock available. In this case, having visibility on sales, the producer supplies the actors downstream.

Regarding inventory checking time, the monitoring of the goods in the warehouse for each actor is carried out by the operators on a pre-established periodic basis. Obviously, this can lead to inefficiencies and generate unfilled orders. However, in the second and third scenarios, the monitoring between the real and the virtual warehouse is carried out in real time by using RFID sensors and the IoT infrastructure that allows information to be sent to the distributed ledger. In this way the actors involved view the product status in real time. In addition, the complete visibility of the stock level allows the producer to make autonomous decisions.

Finally, the documentation sign step in the traditional solution is manual. Very often this phase can generate problems of authenticity and incorrect documentation and consequently the processing time for the monitoring can be longer. Instead, in the second and third scenarios, the documentation management is carried out through a digital certification in which the signatures management is based on public and private keys. This allows for greater safety and speed of delivery operations. Table 7 summarizes the main differences between the three scenarios. The Appendix A shows the data input for each actor and simulation step (see Tables 10, 11, 12, 13 and 14).

**Table 7:** Schematization of comparison between the three different scenarios

	Traditional scenario	Blockchain, Rfid and IoT scenario	VMI and emerging technologies scenario
Order management	Manual order management with tools such as Excel, email, fax, telephone and different IT systems by the players in the supply chain	Automated order management through smart contracts between all the players in the supply chain	Automated order management with smart contract controlled by the producer
Inventory checking time	Manual periodic monitoring of the level of stock in the warehouse carried out by the operators	Real time control and total tracking of products during all phases carried out by RFID sensors	The same of second scenario
Sign documentation time	Paper management of transport documentation	Digital documentation management in a certified way using blockchain	The same of second scenario

## Results

Table 8 shows the results of the three simulation models. The time to order was defined as the time needed to have the required quantity of goods in stock and was evaluated between the producer and the wholesaler. This indicator includes the phases in which the commercial unit of the wholesaler defines the order to be executed until the goods arrive in its warehouse. The first step consists in the analysis of the needs in which the wholesaler's order management department defines the request for goods. Then, there is the order fulfilment phase where the wholesaler’s staff prepares the documentation and contacts for the producer. The order arrives at the producer and the order acceptance and processing step begins. After accepting the order, the producer will contact the delivery company agreeing on times and transport methods. Finally, the order is prepared and shipped on the agreed day and then there will be the transport phase. The last step concerns the checking of the wholesaler regarding the goods supplied. The difference between the traditional scenario and the one with the use of the technology consists in a saving times of approximately 20 h and the percentage variation between scenario 1 and 2 and scenario 1 and 3 is approximately 13%. The lead time of the producer's order preparation can be estimated as the sum of the time required to carry out the warehouse activities. The times considered are:

- **Order management time** is the time taken to evaluate whether to fulfil an order and the time needed to organize the warehouse activities;
- **Picking and checking time** is the time taken to pick up the products needed to complete a shipment batch from the storage area and to carry out the quality check of each product;
- **Pallet packing time** is the time required for packing single product and for creating batches to be loaded into trucks for the shipping.

**Table 8:** Output parameters analysed

	Unit	Traditional Model (Mean)	Blockchain, IoT and RFID Model (Mean)	VMI and emerging technologies model (mean)	%Δ (1–2)	%Δ (1–3)	%Δ (2–3)
Time to order (P-W)	hour	162	142	140	12.63%	13.29%	0.75%
Lead time order preparation (P)	hour	89	80	78	10.64%	12.24%	1.79%
Shipping time (DC)	hour	36	36	36	0.78%	0.83%	0.00%
Consumers (RA)		43,630	43,743	43,804	0.26%	0.40%	0.14%
Unfilled orders (RA)	item	1745	201	120	88.48%	93.14%	40.46%
Service level (RA)	%	96.00%	99.51%	99.73%	3.66%	3.88%	0.22%
Lead Time (RA)	hour	29	17	18	42.20%	40.18%	3.50%
Consumers (RB)		32,871	32,907	32,880	0.11%	0.03%	0.08%
Unfilled orders (RB)	item	1644	99	16	93.98%	99.03%	83.84%
Service level (RB)	%	95.00%	99.70%	99.95%	4.95%	5.21%	0.25%
Lead Time (RB)	hour	34	17	18	48.93%	46.73%	4.31%
Consumers (RC)		21,910	21,912	21,905	0.01%	0.02%	0.03%
Unfilled orders (RC)	item	1205	43	6	96.43%	99.50%	86.05%
Service level (RC)	%	94.50%	99.81%	99.97%	5.62%	5.79%	0.16%
Lead Time (RC)	hour	36	18	18	49.96%	51.16%	2.40%

Also in this case, the time is reduced by about 11% between scenario 1–2 and 12% between scenario 1–3. The improvement in terms of effectiveness and efficiency is due to the time savings for the order management and the real-time control of the products stored in the warehouse.

The shipping time is the time from the departure of the producer's goods until delivery to the wholesaler. It considers three times: first, time for checking goods and bureaucratic procedures for verifying the conformity of the goods, documents loading that will accompany the shipment; then, the transit time is the time it takes for the goods to arrive at their destination; finally, the time for unloading goods and bureaucratic procedures, in which it is necessary to check the conformity of the goods, unloading from transport and signing the documents that will certify the delivery.

Finally, unfilled orders are the customers who have not found the product on the retailers’ shelf, while the service level is the percentage of these dissatisfied consumers. It is shown that the average unfilled orders have decreased between scenario 1 and 2 by 92%, however between scenario 2 and 3 there is a further optimization of about 70%. The last indicator is the supply lead time, which corresponds to the time that elapses between the sending of the retailer's order to the wholesaler upon arrival of the ordered goods. Moreover, the greatest average variation between scenario 1 and scenario 2 is approximately 47% thanks to the automation of various processes.

**Fig. 6:**
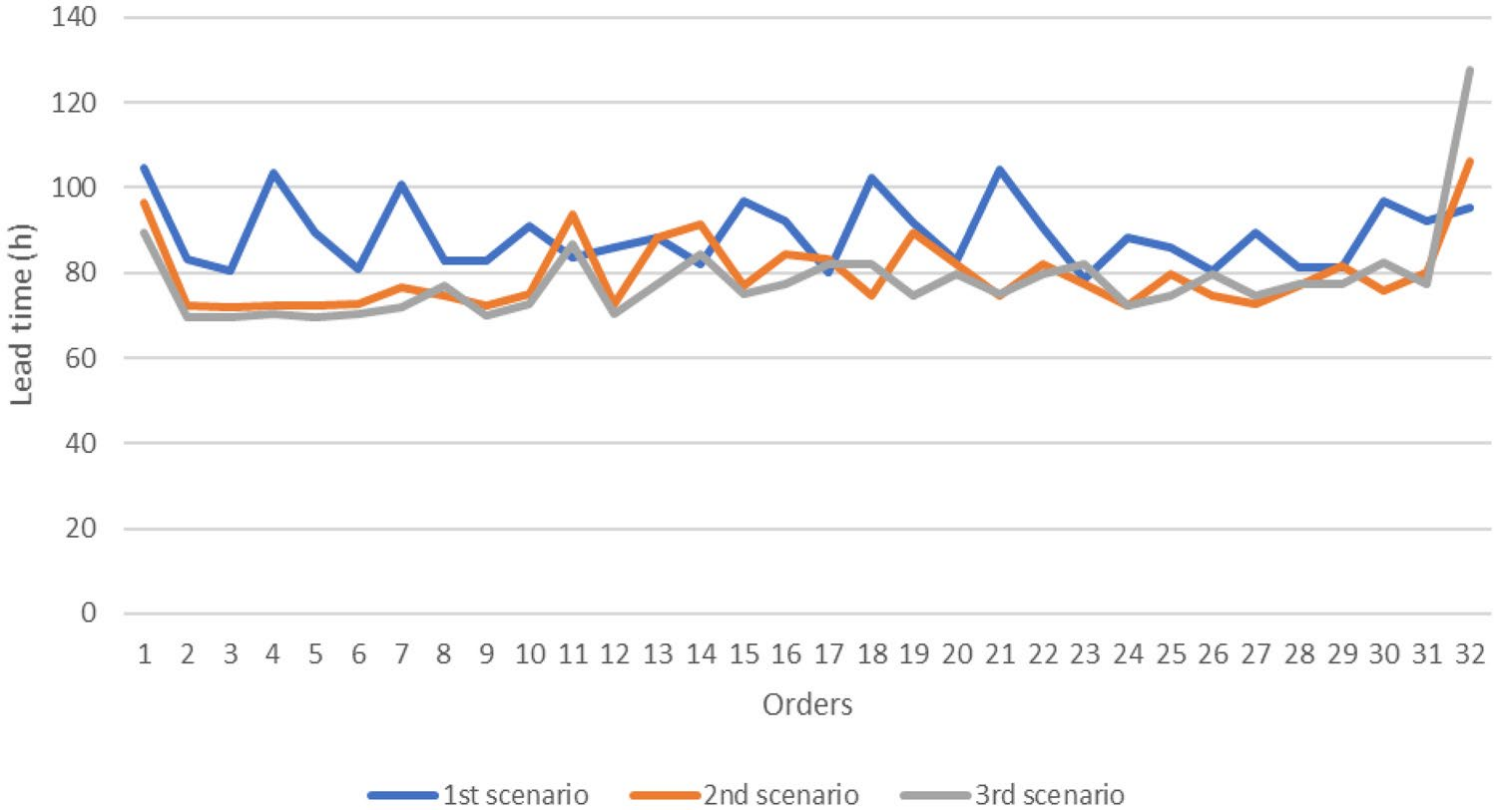
Lead time order preparation for the producer

The graphs of the most significant output parameters of the simulation are presented below: lead time order preparation, time to order (P-W) and unfilled order. The lead time order preparation (Fig. 6) for the producer order is optimized with the introduction of blockchain as it reduces the order management time using smart contracts. In addition, the VMI strategy further reduces this time as the producer can plan their activities in advance.

**Fig. 7:**
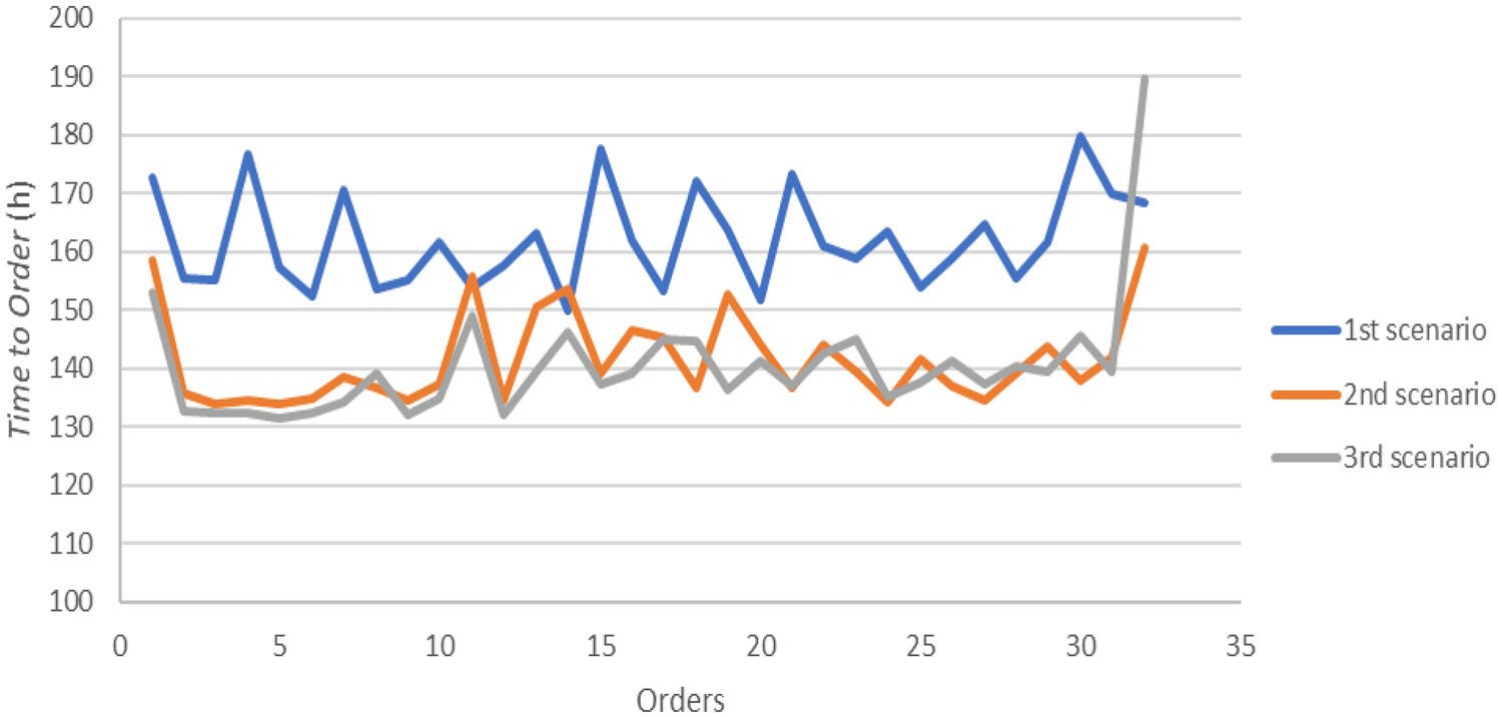
Time to order between the producer and the wholesaler

The values assumed by the time to order for the second and third scenarios are on average lower than the values of the first scenario (Fig. 7). The result is due to the more streamlined and automated activities, making the processes less random, as human work times are reduced. A lower time to order value allow a greater speed of customer service and less probability of stockout occurring. For the producer, the benefits of a reduction in delivery time are twofold. First, faster processes allow for economic savings as the time spent by human resources is less. The second aspect concerns greater customer satisfaction as the goods manage to arrive earlier at their destination having better control and traceability of processes.

**Fig. 8:**
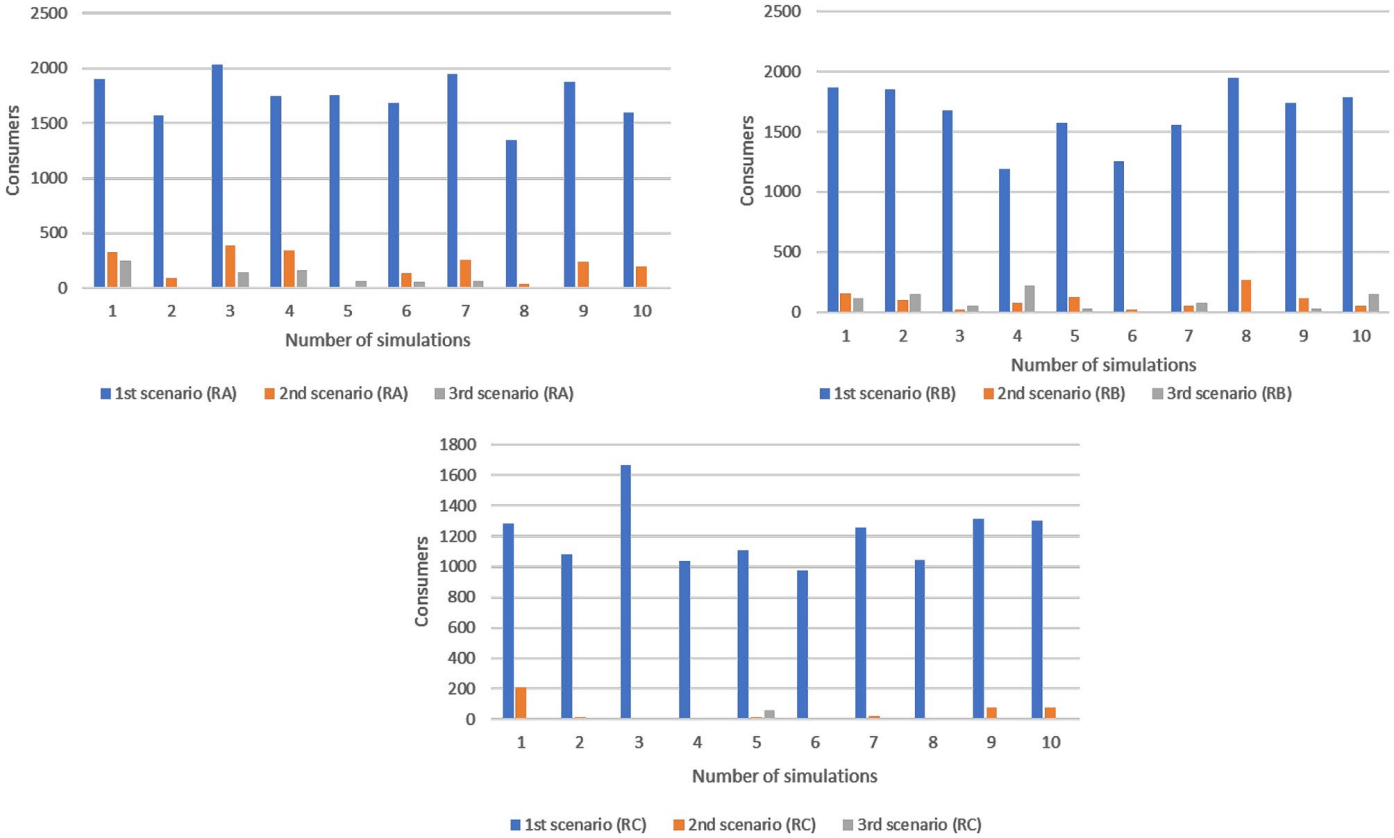
Average unfilled orders among the three retailers

These output parameters optimize the activities within the supply chain, consequently allowing greater satisfaction of retailers' customers. The graph in Fig. 8 shows how on ten simulations the percentage of filled orders in the second and especially in the third scenario has significantly improved.

### Sensitivity analysis

A sensitivity analysis is carried out to consider the influence of structural parameters on the model outputs. There are different types of sensitivity analysis, depending on the target of the analysis (Borgonovo and Plischke 2016). In particular, factor prioritization was chosen to study the influence of the different parameters of the model. Three structural parameters were considered: delivery time of DC, % product purchase and ageing time. These parameters were varied from -20% to + 20% in four steps. The variations of these parameters carry out evaluations on the unfilled orders. Table 9 shows the values of such variations.

**Table 9:** Results of the sensitivity analysis considering the unfilled orders variations

Parameter	Value	%∆1–2	%∆1–3	%∆2–3
Delivery time (DC)	-20%	81%	86%	24%
	-10%	78%	78%	0%
	10%	69%	86%	54%
	20%	77%	78%	8%
%Product purchase	-20%	99%	100%	100%
	-10%	90%	91%	10%
	10%	31%	37%	8%
	20%	17%	24%	9%
Ageing time	-20%	89%	100%	100%
	-10%	84%	89%	31%
	10%	96%	97%	20%
	20%	81%	83%	12%

Results show that the variations in the percentage of product purchase have a significant influence on fulfilled orders. In particular, the higher the percentage of products purchase, the greater the reactivity to satisfy the demand for the second and third scenarios. Scenarios 2 and 3 show how the technologies and VMI strategy significantly limit these problems. A solution with blockchain and smart contract enables the optimization of the supply chain allowing an improvement in the resilience of the supply chain. However, the variation in timing parameters such as the delivery time of DC and the ageing time of the cheese do not show any significant variation for the unfilled orders.

## Discussion

This section deepens the research results to answer the two questions that guided this work and provides some research implications.

### On which supply chain operations can blockchain, IoT, RFID and smart contract derive operational time benefits for organizations?

The study showed the impact in terms of time performances that blockchain, connected to other technologies, has on supply chain operations from a numerical point of view. Starting from previous studies combining VMI and blockchain (Dasaklis and Casino 2019; Guggenberger et al. 2020; Omar et al. 2020), the study derived the time impacts on the Parmigiano Reggiano supply chain. The results in Table 8 show the variations in terms of time performance. The time advantage of the second scenario mainly depends on the technologies that capture the data, i.e. RFID and IoT. In addition, time is reduced because the needs analysis process is optimized by the presence of real-time inventory tracking systems. In this way, the order management unit has better visibility of the inventory in its physical warehouse by consulting the distributed ledger. Consequently, by combining continuous monitoring and exploiting the VMI strategy, the optimization of time and the reduction of unfilled orders is further improved. However, the difference between the time to order and lead time order preparation variables between the three scenarios have not drastically changed. The variation among the three scenarios ranges between 10 and 13%. Blockchain technology and the smart contracts facilitate tracking systems, visibility of the entire supply chain and allow greater trust and collaboration between partners. The VMI strategy, which may be unusable when there are opportunistic behaviours between partners, is implementable and guarantees the achievement of the results since the blockchain guarantees the concept of trust as the transactions within it are safe and immutable. As widely recognized in the literature, VMI regulates the frequency of purchased orders because the seller has a complete visibility of the downstream demand (Taleizadeh et al. 2015). Thus, the bullwhip effect is mitigated by reducing the variance of demand. Furthermore, the benefits of shorter delivery times are twofold for the producer. On the one hand, faster processes allow for economic savings as the time spent by human resources is less. On the other hand, there is greater customer satisfaction as goods arrive earlier at their destination with full process control and traceability. The use of these tools can increase the company's reputation towards the final market.

Note that shipping time is almost unchanged because it is mainly based on material flow operations. Indeed, blockchain impacts on information flows but not on material ones. The order management is more automated and improves time performances for each operation. However, for real optimization it is necessary to equip the other areas such as inventory and logistics with technologies such as IoT and RFID for data capture. As for the output parameters on each retailer, it is evident that the capture of information in real time and implementing the VMI strategy carries out benefits in terms of time advantages and customer satisfaction. As confirmed by the sensitivity analysis, by varying the percentage of product purchase, the scenarios with emerging technologies and the VMI strategy are more reactive to meet demand and make the supply chain resilient. This can be explained by the fact that in the traditional scenario relationships among organizations are performed by neighbouring players and planning is done only on the historical purchase data that each actor receives from their downstream counterparts. For the scenario with both emerging technologies and the use of VMI, the forecasts are more accurate since the data are updated daily on the blockchain and shared among all the players. It is interesting to note the percentage differences between the second and third scenarios (%Δ2-3) in Table 8. The only variables significantly changed in the third scenario compared to the second one are the reduction in unfilled orders of the three retailers which vary from 40 to 86%. The other variables, such as time to order, lead time order preparation and lead time to the retailers in the third scenario do not differ significantly from the second. This implies that emerging technologies enable time reduction on some activities. The adoption of the VMI strategy, enabled by these tools, mainly strengthens the customer satisfaction parameter by reducing the unfilled orders.

The orders planning carried out by the producer improves the overall efficiency of the supply chain thanks to the visibility of downstream demand. The use of blockchain changes the operations processes and organizational models of companies allowing for better data sharing. Therefore, the innovativeness of the model presented consists in measuring *ex-ante* the impacts that integration of different technologies can introduce within the Parmigiano Reggiano supply chain. Blockchain can be considered as an enabling tool for more effective and efficient operations management. The results demonstrate that blockchain technology is a cost-effective tool for overcoming the problems of collaboration and trust in a supply chain and for minimizing the negative impacts of information asymmetry at the supply chain level.

### What are the benefits for each participant for supply chain operations in the various scenarios considered?

The benefits that the second and the third scenarios bring to each participant regard the exchange of information on a single platform among the players. The inaccuracies of the specifications and the lack of clarity that arises in the first scenario are reduced by using blockchain and smart contracts. For example, non-standard order acquisition systems such as emails and phone calls can generate errors and further waste of time by increasing order fulfilment times for each actor. The timing for the order management of the three scenarios is different. With the information sharing of the distributed ledger, there is greater traceability of orders, better visibility for participants and consequently greater trust in operations without the use of other intermediaries. Employing a single platform for the transactions exchange, the orders receipt and processing are carried out in a standardized way with the smart contracts.

In the second and the third scenarios, RFID sensors, IoT infrastructure and blockchain are implemented within the producer and wholesaler’s warehouse and during the shipment. Therefore, this system architecture allows to acquire the stock level data in real time through RFID sensors, transfer the data on the blockchain using the IoT infrastructure and finally record them permanently and securely within the blockchain. This configuration saves time for the order management unit and reduces the time it takes for an operator to check, identify and record the position of the product in the warehouse. Finally, retailers request the goods via the blockchain platform by activating a smart contract. In this case, retailers are guaranteed the quality of the goods thanks to the complete traceability and visibility provided by the blockchain. The second and third scenarios solve the communication problems among the actors, reduce the potential waste of time due to human error and the presence of unnecessary bureaucratic activities. In particular, the VMI strategy of the third scenario reduces the workload of the actors downstream of the producer, guaranteeing greater flexibility in satisfying the final consumers.

### Research implications

This is one of the first studies that clarifies and shows *ex-ante* the impacts in terms of time performances on operations that the integration of emerging technologies and the VMI strategy can have on supply chains. The second and third scenarios show how information exchange, control and monitoring on a shared platform can reduce procurement lead times and unfilled order. The research provides various insights into how the supply chains can be reorganized in different areas with the introduction of emerging technologies. It highlights the role of blockchain as an enabler of the VMI strategy for the operations management.

The study provides a first benchmark to managers and practitioners regarding the contribution of new technologies within supply chains and how these emerging technologies can be employed in supply chain operations. Real and pilot cases are currently in development in the real world. The use of simulation as a research tool allows to compare and analyse different alternatives in the absence of a real system model. The study investigates the advantages of applying these technologies by reducing the potential implementation risks through the analysis of quantitative parameters. Models that employ emerging technologies strengthen collaborative relationships and trust between partners as well as automate some operations by increasing the reputation among each participant in the network. The study aims to incentivize the adoption of these technologies which is still slow as the potential benefits are not clear. However, several issues remain open, for example, how to integrate these technologies with other IT systems or how to reduce the knowledge and technical skills gap to properly manage these emerging tools.

## Conclusions

The visibility of the chain is one of the problems that most afflicts modern chains. Most of the solutions present are of the one up-one down visibility type, i.e. the partners manage the relationships with the closest players upstream and downstream. However, an end-to-end visibility involves all the actors in the supply chain so that anyone could understand the external dynamics. To date the single source of truth is used, based on the involvement of a trusted third party who takes care of managing information in the supply chain. Alternatively, whoever manages the information can be a leader in the supply chain. However, these two solutions are not always achievable because, on the one hand, the third party may not exist, or the leader company does not want to manage the information of the other stakeholders. Furthermore, the players in the supply chain may have IT systems that are difficult to integrate with each other. Blockchain, being a distributed ledger, converts the concept of single source of truth into common source of truth and allowing a shared and unitary vision of reality. Collaborative environments based on trust and information sharing can be successful using blockchain technology as an intermediary. Several properties of blockchains are beneficial: the decentralized nature of data storage, data validation, immutability and transparency. The process of sharing information and data is more resilient as there is no single point of failure. This will lead to greater transaction confidence and mitigate cybersecurity risks.

This article aims to investigate different scenarios of the Parmigiano Reggiano supply chain with the application of integrated technologies and the combination of the VMI strategy. The work also conducts an in-depth analysis on the use of blockchain as an enabling factor for the VMI strategy. In the three proposed scenarios, the effects that the combined use of smart contracts, IoT blockchains, RFID and VMI have on the efficiency and effectiveness of the considered supply chain are studied. The results show how the capture of information in real time and the total visibility of the chain with the distributed ledger has a positive improvement in terms of time performance and fulfilled orders. The importance of building new organizational models is highlighted, considering the combined use of these techniques and technologies to ensure the achievement of better results.

However, further theoretical, empirical and quantitative studies are needed on the actual benefit of the connected use of these technologies. First, a necessary assessment should be carried out on the cost analysis of the entire technological infrastructure and the impact it has on each actor in the supply chain. In addition, a network with a larger number of participants should be considered in order to evaluate the technological performance of the blockchain in terms of scalability, throughput, storage and latency. Further investigation would be needed on how these technological, cost and time performance together could impact on entire supply chains. Moreover, it is necessary to consider different scenarios with different conditions such as international logistics, the risks of disruption events due to catastrophic phenomena such as the covid-19 pandemic, in order to better understand the real advantages and challenges of these new solutions. It is necessary to investigate how blockchain technology could enable other areas, such as humanitarian activities, to improve collaboration management, information sharing and disruption event management for catastrophic events for a more resilient supply chain.

## Data Availability

The study did not report any data.

## Appendix A. Data input of the simulation scenarios

**Table 10:** Data Input (P)

		Traditional model	Blockchain, IoT and RFID model	VMI and emerging technologies model
		Unit	Unit	Unit
Production time		21 Days	21 Days	21 Days
Ageing time		12 Months	12 Months	12 Months
Storing time	Triangular distribution—Minimum	18 s	18 s	18 s
	Mode	30 s	30 s	30 s
	Maximum	1 min-	1 min	1 min
Picking and checking time	Triangular distribution—Minimum	1 min	1 min	1 min
	Mode	1,5 min	1,5 min	1,5 min
	Maximum	2 min	2 min	2 min
Packing pallet time	Triangular distribution—Minimum	50 s	50 s	50 s
	Mode	1 min	1 min	1 min
	Maximum	1,20 min	1,20 min	1,20 min

**Table 11:** Data Input (DC)

Checking pallet time	Normal distribution—Mean	15 min	15 min	15 min
	SD	1 min	1 min	1 min
Loading pallet time	Normal distribution—Mean	40 min	40 min	40 min
	SD	2 min	2 min	2 min
**Sign documentation time (P—DC)**	**Normal distribution—Mean**	**5 min**	**5 s**	**5 s**
	**SD**	**1 min**	**0.5 s**	**0,5 s**
Shipping time by truck	Triangular distribution—Minimum	4 h	4 h	4 h
	Mode	4 h, 20 min	4 h, 20 min	4 h, 20 min
	Maximum	4 h, 40 min	4 h, 40 min	4 h, 40 min
Shipping time by ship	Normal distribution—Mean	29 h	29 h	29 h
	SD	30 min	30 min	30 min
Shipping time by truck	Normal distribution—Mean	2 h	2 h	2 h
	SD	5 min	5 min	5 min

**Table 12:** Data Input (W)

Checking pallet time	Normal distribution—Mean	15 min	15 min	15 min
	SD	1 min	1 min	1 min
Unloading pallet time	Normal distribution—Mean	40 min	40 min	40 min
	SD	2 min	2 min	2 min
**Sign documentation time (DC—W)**	**Normal distribution—Mean**	**5 min**	**5 s**	**5 s**
	**SD**	**1 min**	**0.5 s**	**0.5 s**
Storing time	Triangular distribution—Minimum	4 min	4 min	4 min
	Mode	5 min	5 min	5 min
	Maximum	7 min	7 min	7 min
Picking and packing time	Triangular distribution—Minimum	4 min	4 min	4 min
	Mode	5 min	5 min	5 min
	Maximum	7 min	7 min	7 min

**Table 13:** Data Input (R)

Shipping time by truck (A)	Normal distribution—Mean	30 min	30 min	30 min
	SD	5 min	5 min	5 min
Shipping time by truck (B)	Normal distribution—Mean	50 min	50 min	50 min
	SD	4 min	4 min	4 min
Shipping time by truck (C)	Normal distribution—Mean	60 min	60 min	60 min
	SD	5 min	5 min	5 min
Customers (A)	Interarrival time (Exponential distribution)	1600 customers/day	1600 customers/day	1600 customers/day
Customers (B)		1200 customers/day	1200 customers/day	1200 customers/day
Customers (C)		800 customers/day	800 customers/day	800 customers/day
% Product purchase	Triangular distribution—Minimum	3%	3%	3%
	Mode	5%	5%	5%
	Maximum	7%	7%	7%

**Table 14:** Data Flow Input

Order management (P)	Normal distribution—Mean	1 h	5 min	5 min
	**SD**	**2 min**	**30 s**	**30 s**
**Order management (DC)**	**Normal distribution—Mean**	**1 h, 30 min**	**10 min**	**10 min**
	**SD**	**2 min**	**30 s**	**30 s**
**Order management (W)**	**Normal distribution—Mean**	**1 h, 30 min**	**5 min**	
	**SD**	**2 min**	**30 s**	
**Order management (R)**	**Normal distribution—Mean**	**1 h**	**5 min**	
	**SD**	**2 min**	**30 s**	
